# Development of an sRNA-mediated conditional knockdown system for *Chlamydia trachomatis*

**DOI:** 10.1128/mbio.02545-24

**Published:** 2024-12-13

**Authors:** Janina Ehses, Kevin Wang, Asha Densi, Cuper Ramirez, Ming Tan, Christine Sütterlin

**Affiliations:** 1 Department of Developmental and Cell Biology, University of California, Irvine, California, USA; 2 Department of Microbiology and Molecular Genetics, University of California, Irvine, California, USA; 3 Department of Medicine, University of California, Irvine, California, USA; Weill Cornell Medicine, New York, New York, USA

**Keywords:** engineered sRNA, protein depletion, operon, essential genes, genetics

## Abstract

We describe a new *Chlamydia trachomatis* protein depletion method that uses an engineered small RNA (sRNA) to inhibit translation of a target gene. In proof-of-principle experiments, we induced functional knockdown of IncA, a fusion-mediating inclusion membrane protein, as shown with Western blots, loss of IncA staining at the inclusion membrane, and production of multiple chlamydial inclusions within an infected cell. These effects were titratable and reversible. To test for polar effects, we separately targeted the inclusion membrane proteins IncE and IncG, which are expressed from the *incDEFG* operon. Knockdown of IncE caused loss of IncE and its interacting host protein SNX6 at the inclusion membrane, without affecting IncG protein levels. Similarly, IncG knockdown significantly reduced IncG levels and prevented recruitment of its interacting host protein 14-3-3β, without altering IncE protein levels. These data provide the first genetic evidence that IncE and IncG are necessary for the recruitment of SNX6 and 14-3-3β, respectively, demonstrating the value of this knockdown approach. We also successfully depleted the major chlamydial surface protein, major outer membrane protein (MOMP), which is encoded by a likely essential gene that has not been previously disrupted or knocked down. MOMP knockdown caused severe defects in bacterial morphology and progeny production. Thus, our sRNA-based approach has broad potential as a conditional knockdown method for studying the function of *C. trachomatis* genes, including essential genes and genes in an operon.

**IMPORTANCE:**

We describe a new method to reduce protein levels of a selected gene in the pathogenic bacterium *Chlamydia trachomatis*. This approach utilizes an engineered small RNA (sRNA) to inhibit translation of the mRNA for a target gene and produced inducible and reversible protein knockdown. Our method successfully knocked down four proteins, including a likely essential gene and individual genes in an operon, without altering protein levels of a neighboring gene. This conditional knockdown method will be useful for studying the function of genes in *Chlamydia*. It also has the potential to be applied to other obligate intracellular bacteria, including *Rickettsia* and *Coxiella*.

## INTRODUCTION

*Chlamydia trachomatis* is the most common cause of bacterial sexually transmitted disease (1). Each year, more than 1.5 million new cases are reported in the United States, which is more than all other reported notifiable infections combined (2). Almost all of these *C. trachomatis* infections are urogenital infections, and their long-term sequelae primarily affect female reproductive health, including increased rates of infertility and ectopic pregnancy (1). In less developed countries, *C. trachomatis* also causes an eye disease called trachoma, which is the most common infectious etiology of preventable blindness (1).

*C. trachomatis* is an intracellular bacterium that requires an epithelial host cell for its replication. It has a unique 48–72 h developmental cycle in which there is conversion between two specialized forms of the bacterium (3, 4). An infectious form, called the elementary body (EB), binds and enters the host cell. Within the membrane-bound chlamydial inclusion, the EB differentiates into a reticulate body (RB), which is the metabolically active, dividing form. RBs undergo multiple rounds of replication to expand the bacterial population and then asynchronously convert into EBs for release to infect new host cells.

Like other obligate intracellular bacteria, *C. trachomatis* has a small genome compared to free-living bacteria (5). Only 904 genes are annotated for *C. trachomatis*, which is approximately 20% of the 4,400 genes in *Escherichia coli* (6). Genes are believed to have been lost because their functions are no longer needed or have been replaced by host factors, in a process known as reductive evolution (5). As a consequence, a high proportion of the retained genes are likely to be essential and cannot be studied with traditional gene disruption methods, unless paired with a conditional rescue scheme (7, 8). More than 60% of *C. trachomatis* genes are of unknown function (6).

The function of essential genes can be investigated with inducible knockdown methods that deplete protein levels of a targeted gene. For *C. trachomatis*, the only knockdown method that has been developed is clustered regularly interspaced short palindromic repeats interference (CRISPRi), which has been used to repress the expression of targeted genes (9–13). This method utilizes a guide RNA (sgRNA) to direct a catalytically inactive form of a Cas endonuclease (dCas9 or dCas12) to the promoter of the target gene to block transcription (14, 15). However, this promoter-based approach is not suitable for selectively targeting a gene in an operon because all genes in the operon are co-transcribed from the same promoter (10).

An alternative knockdown strategy is to inhibit translation rather than transcription because prokaryotic genes are commonly translated from their own ribosome binding site (RBS) and start codon (16). Such an approach is based on the ability of a non-coding RNA called a small RNA (sRNA) to bind and block ribosome recruitment to the RBS in a sequence-dependent manner, thereby preventing translation (17). For targeted knockdown, the sRNA is engineered to recognize the RBS in the 5′ untranslated region of the gene of interest. This sRNA-based approach has been successfully used as a conditional knockdown method in model bacteria such as *E. coli* (18–20) without causing polar effects (21). sRNA-mediated regulation is titratable because it depends on the stoichiometry between the levels of the sRNA and its mRNA target (22). However, an important consideration is that *Chlamydia* differs from other bacteria in lacking Hfq (23), which is a conserved RNA chaperone that facilitates base pairing between an sRNA and its mRNA targets and also recruits effector proteins. Thus, it is not known if this sRNA-based knockdown approach can be applied to *C. trachomatis* and other *Chlamydia* spp.

In this manuscript, we describe a conditional knockdown method that is new to *Chlamydia*, and which utilizes an engineered sRNA to deplete protein levels of a target gene. We separately knocked down four *C. trachomatis* proteins by replacing the target recognition site of the *C. trachomatis* sRNA CtrR3 (24) with sequences antisense to the RBS region of each target mRNA. We also investigated if this novel method can selectively target a gene in an operon without causing polar effects and whether it can knock down an essential gene.

## RESULTS

### Knockdown of IncA with an engineered sRNA

In proof-of-principle studies, we tested if we could reprogram an sRNA to knockdown a well-studied, non-essential *C. trachomatis* protein. This knockdown method is based on the canonical mechanism by which a bacterial sRNA recognizes and binds a target mRNA and interferes with its translation (18–20). We chose to reprogram CtrR3, a *C. trachomatis* sRNA that has a simple predicted secondary structure, with a long stem and a single loop (25, 26). In addition, CtrR3 is constitutively expressed during the *Chlamydia* developmental cycle (24) and is therefore less likely to be controlled by post-transcriptional mechanisms that could alter the levels of our engineered CtrR3. Our previous analysis showed that a two-nucleotide substitution in the loop sequence abrogated the progeny defect of CtrR3 overexpression, providing evidence that the CtrR3 target recognition sequence is contained within this loop and that overexpression of the CtrR3 backbone is not deleterious (24). Thus, this loop was the logical site to place our target sequence. For this and subsequent engineered versions of CtrR3, we routinely checked that the predicted secondary structure was not altered by the inserted target sequence.

**Fig 1:**
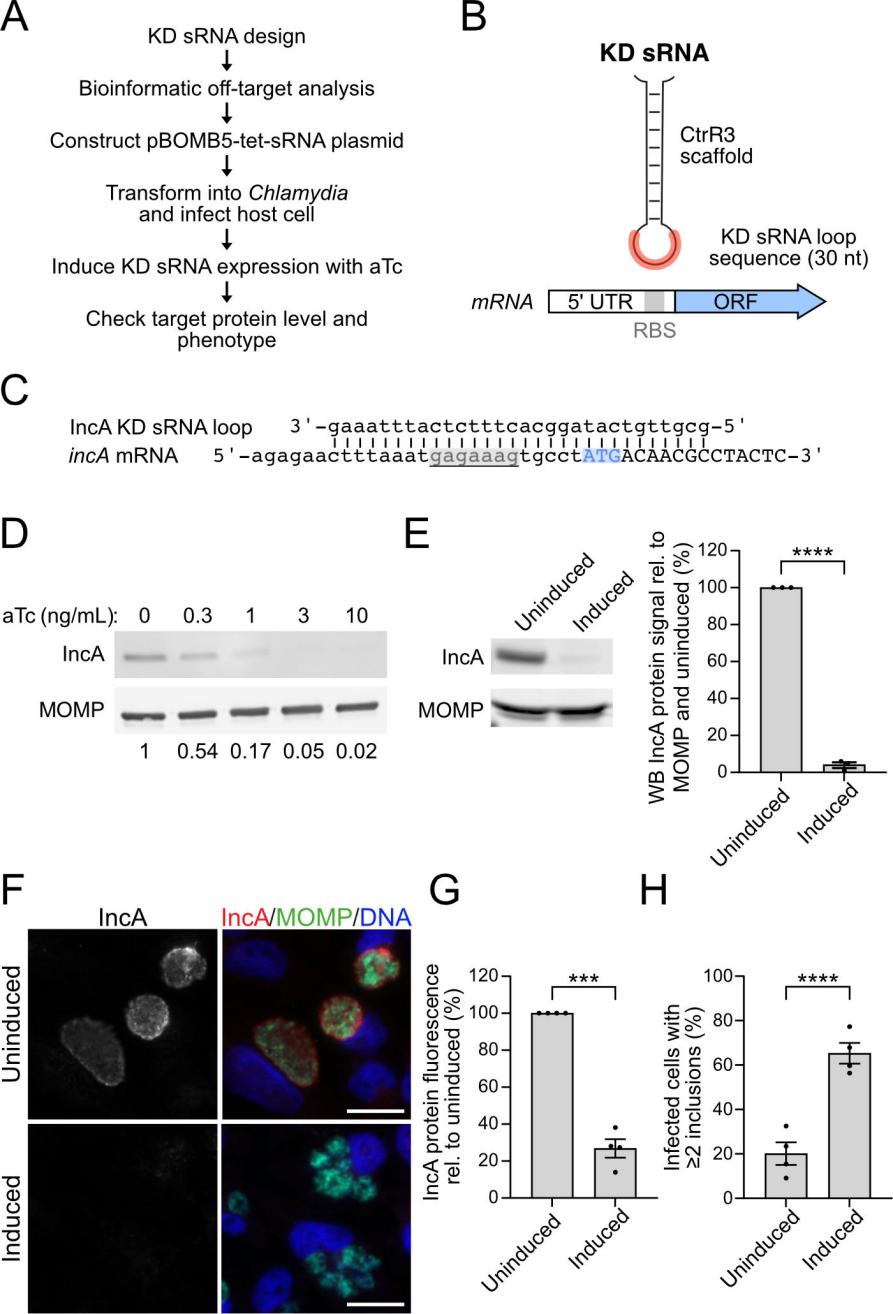
Engineered sRNA-mediated knockdown (KD) of IncA in *Chlamydia*. (**A**) Scheme of the KD sRNA approach. (**B**) Design of the engineered sRNA, which uses *C. trachomatis* CtrR3 as the sRNA backbone, but with its single main loop replaced by a 30-nucleotide targeting sequence (shown in red) that is antisense to a region containing the start codon and the RBS (shown in gray) of the mRNA for the target gene. (**C**) Alignment of the IncA KD sRNA targeting sequence and *incA* mRNA. The potential RBS of *incA* is underlined and highlighted in gray, and the start codon is highlighted in blue. (**D**) Western blot of total cell lysates from HeLa cells infected with the IncA KD transformant, treated with different concentrations of aTc at 1 hpi and harvested at 24 hpi. Normalized values (to MOMP and uninduced) are shown below the Western blot (*n* = 1). (**E**) Western Blot analysis of total cell lysates from HeLa cells infected with the IncA KD transformant, either uninduced or induced with 3 ng/mL of aTc at 1 hours post infection (hpi) and harvested at 24 hpi. IncA and MOMP (loading control) protein levels were detected with antibodies raised against these respective proteins. A quantification of the IncA signal, normalized as in panel **D** is also shown. (**F**) Immunofluorescence images of HeLa cells infected with the IncA KD transformant, either uninduced or induced with 3 ng/mL of aTc at 1 hpi and stained at 24 hpi with antibodies to IncA (red) and MOMP (green). DNA was visualized with Hoechst 33342 and is shown in blue. Scale bar is 20 µm. (**G**) Quantification of IncA fluorescence intensity for whole inclusions. (**H**) The percentage of infected cells with multiple inclusions is shown. For panel **G** and **H**, ≥150 inclusions were analyzed per replicate and condition. The data are shown as mean ± SEM from ≥3 independent biological and technical replicates. Unpaired, two-tailed Student’s *t*-test with Welch’s correction (**E, G**) and paired, two-tailed Student’s *t*-test (**F**) with ***P < 0.001 and ****P < 0.0001. aTc, anhydrotetracycline; WB, Western blot; hpi, hours post infection; KD, knockdown; ORF, open reading frame; UTR, untranslated region.

The overall experimental strategy for our knockdown studies is shown in Fig. 1A. For our initial studies, we attempted to knockdown IncA, an inclusion membrane protein with a deletion phenotype of multiple unfused inclusions at a high multiplicity of infection (MOI) (27, 28). We replaced the single main loop of CtrR3 (Fig. S1A) with a 30-nucleotide sequence antisense to the 5′ region of *incA* mRNA containing its putative RBS and start codon (Fig. 1B; Fig. S1B). We chose this length of targeting sequence because a shorter sequence might not produce sufficient base pairing with the mRNA target, while a longer sequence could increase off-target effects from base pairing to other mRNAs. These considerations were informed by design rules developed for *E. coli* antisense RNAs (29). Our sequence design also included bioinformatic analysis with the sRNA target identification tool TargetRNA3 (30) to select a sequence with the fewest potential off-target binding sites in the *C. trachomatis* genome (Fig. 1C). The IncA KD sRNA was cloned into the pBOMB5-tet-CtrR3 plasmid, which we previously used to overexpress the CtrR3 sRNA (Fig. S1C) (24), and transformed into *C. trachomatis*. This transformant was then used to infect HeLa cells at a relatively high MOI of 5 to be able to detect the multi-inclusion phenotype of IncA depletion.

Induction of IncA KD sRNA expression with anhydrotetracycline (aTc) depleted IncA protein levels at 24 hours post infection (hpi) in a concentration-dependent manner (Fig. 1D). aTc, 3 ng/mL (6.5 nM), produced a 95% reduction in IncA protein levels by Western blot analysis (Fig. 1D and E) and decreased IncA levels at the inclusion membrane to 27 ± 4% of uninduced control cells, as measured by immunofluorescence microscopy (Fig. 1F and G). Our knockdown method is likely to be specific for IncA because it did not alter levels of the chlamydial surface protein major outer membrane protein (MOMP) by Western blot or immunofluorescence (Fig. 1D and F) as an indicator of normal progression of the infection. Transcript levels for *incA*, but not *euo*, were reduced threefold by RT-qPCR (Fig. S1D). As expected, induction of the IncA KD sRNA produced multiple inclusions in 65% ± 3% of infected cells, compared to 20 ± 4% of uninduced control cells (Fig. 1F and H), and no defect in the production of infectious progeny production (Fig. S2A). These results are consistent with the published phenotypes reported for an IncA deletion mutant (31) and demonstrate that that our engineered sRNA produced a functional knockdown of IncA (28, 32).

### IncA knockdown complementation and reversibility

**Fig 2:**
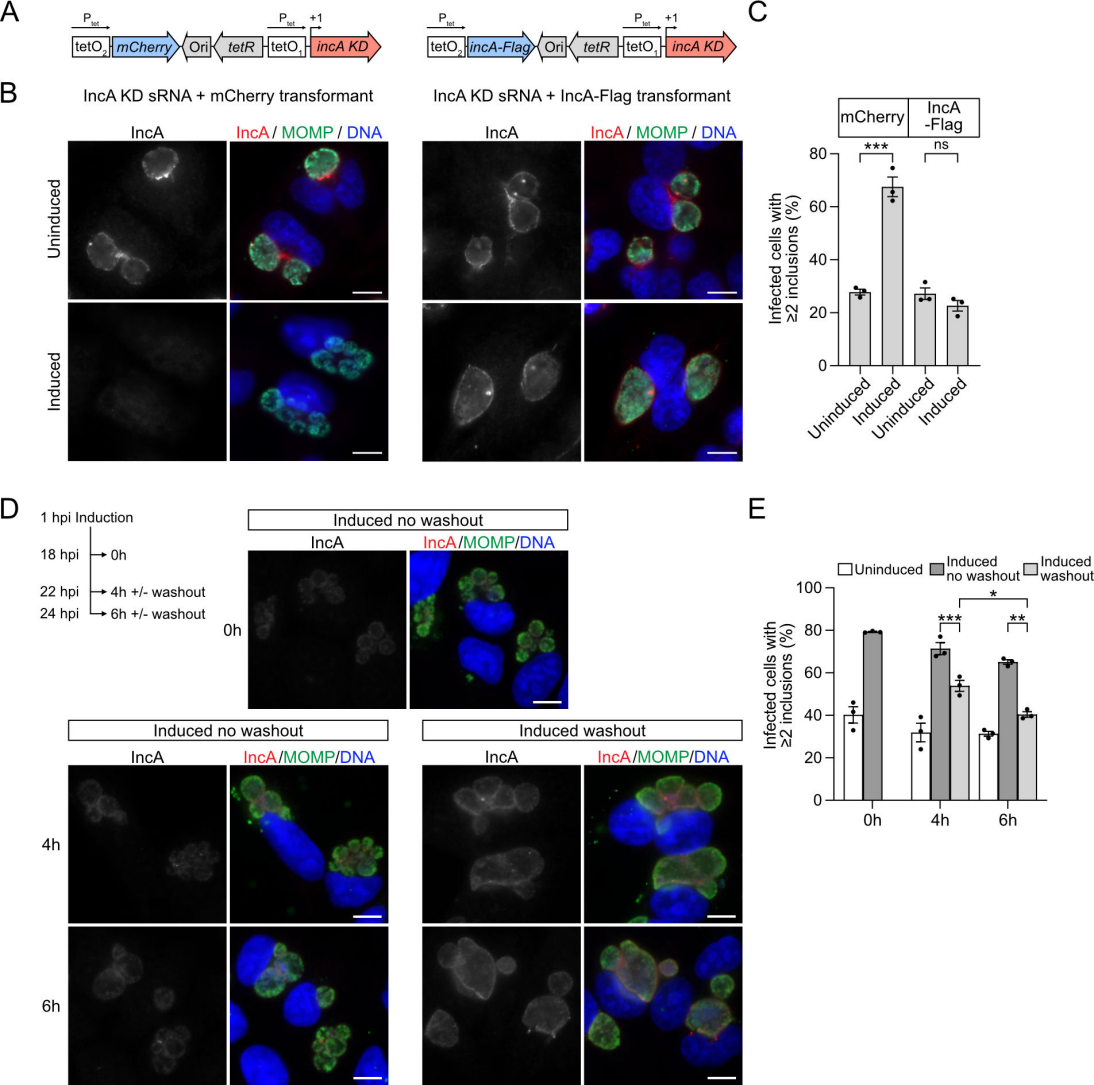
IncA knockdown can be rescued by complementation and is reversible. (**A**) Diagram of control and IncA complementation plasmids containing the tet-inducible IncA knockdown cassette and sequences encoding either mCherry as a negative control (left) or IncA-Flag (right). Expression of mCherry and IncA-Flag is driven by tetO_2_, a weaker tet-promoter from the pASKtet-GFP-L2 plasmid. (**B**) Immunofluorescence images of HeLa cells infected with *C. trachomatis* transformants expressing either IncA KD sRNA + mCherry (left panel) or IncA KD sRNA + IncA Flag (right panel), uninduced or induced with 3 ng/mL of aTc at 1 hpi and stained at 24 hpi with antibodies to IncA (red) and MOMP (green). (**C**) The percentage of cells with multiple inclusions from the experiment in panel **B** is shown. (**D**) Experimental scheme and immunofluorescence images of HeLa cells infected with the IncA KD transformant, induced with 3 ng/mL of aTc at 1 hpi and stained at 18, 22, and 24 hpi with antibodies to IncA (red) and MOMP (green). For washout conditions, aTc-containing media was replaced by regular growth media without aTc at 18 hpi. (**E**) Quantification of the multiple inclusion phenotype of the experiment in panel **D**. Data are shown as mean ± SEM from three independent biological replicates, from analysis of ≥80 cells per replicate and condition. One-way ANOVA with Tukey’s multiple comparisons test (**C**) and multiple paired, two-tailed Student’s *t*-test (**E**) with *P < 0.05, **P < 0.01 and ***P < 0.001; ns, not significant. For all immunofluorescence images, DNA was visualized with Hoechst 33342 and is shown in blue. Scale bar is 10 µm. hpi, hours post infection; h, hours; KD, knockdown.

We complemented the IncA knockdown by expressing a Flag-tagged *incA* gene from its own tet promoter (Fig. 2A). The complementing *incA* allele and the KD sRNA were included on the same plasmid because only a single plasmid can be stably maintained in *C. trachomatis* with current methods (33). Furthermore, the complementing *incA-flag* was designed to be resistant to the KD sRNA by replacing the native RBS with the RBS from the pASKtet-GFP-L2 plasmid, which is not complementary to the KD targeting sequence. Simultaneous induction of IncA KD sRNA and IncA-Flag with 3 ng/mL of aTc restored IncA at the inclusion membrane, as shown by staining with anti-IncA (Fig. 2B) and anti-Flag antibodies (Fig. S2B) and rescued the multiple inclusion phenotype (Fig. 2C). There was no recovery of IncA staining with a control transformant expressing mCherry instead of IncA-Flag (Fig. 2A and B). These data provide evidence that our sRNA method depleted IncA protein and caused the IncA loss-of-function phenotype in a specific manner.

To examine if IncA knockdown is reversible, we performed a washout experiment in which the inducing agent aTc was added at 1 hpi, followed by a medium exchange at 18 hpi to remove the inducer. IncA staining at the inclusion membrane was restored by 4 h after washout (Fig. 2D). In addition, the prevalence of infected cells with multiple inclusions was reduced to 54% ± 2% and 40% ± 1% by 4 and 6 h after washout, respectively, compared to 79% ± 1% prior to washout (Fig. 2D and E). In contrast, there was no recovery of IncA levels nor correction of the multiple inclusion phenotype in a “no washout” control (Fig. 2D and E). Also, IncA levels remained stable over time in our uninduced control sample (Fig. S2C and E). These results demonstrate that the duration of IncA protein depletion could be controlled and that functional knockdown could be reversed by removing the inducing agent from the cell culture medium.

### Genes in an operon can be knocked down without polar effects

**Fig 3:**
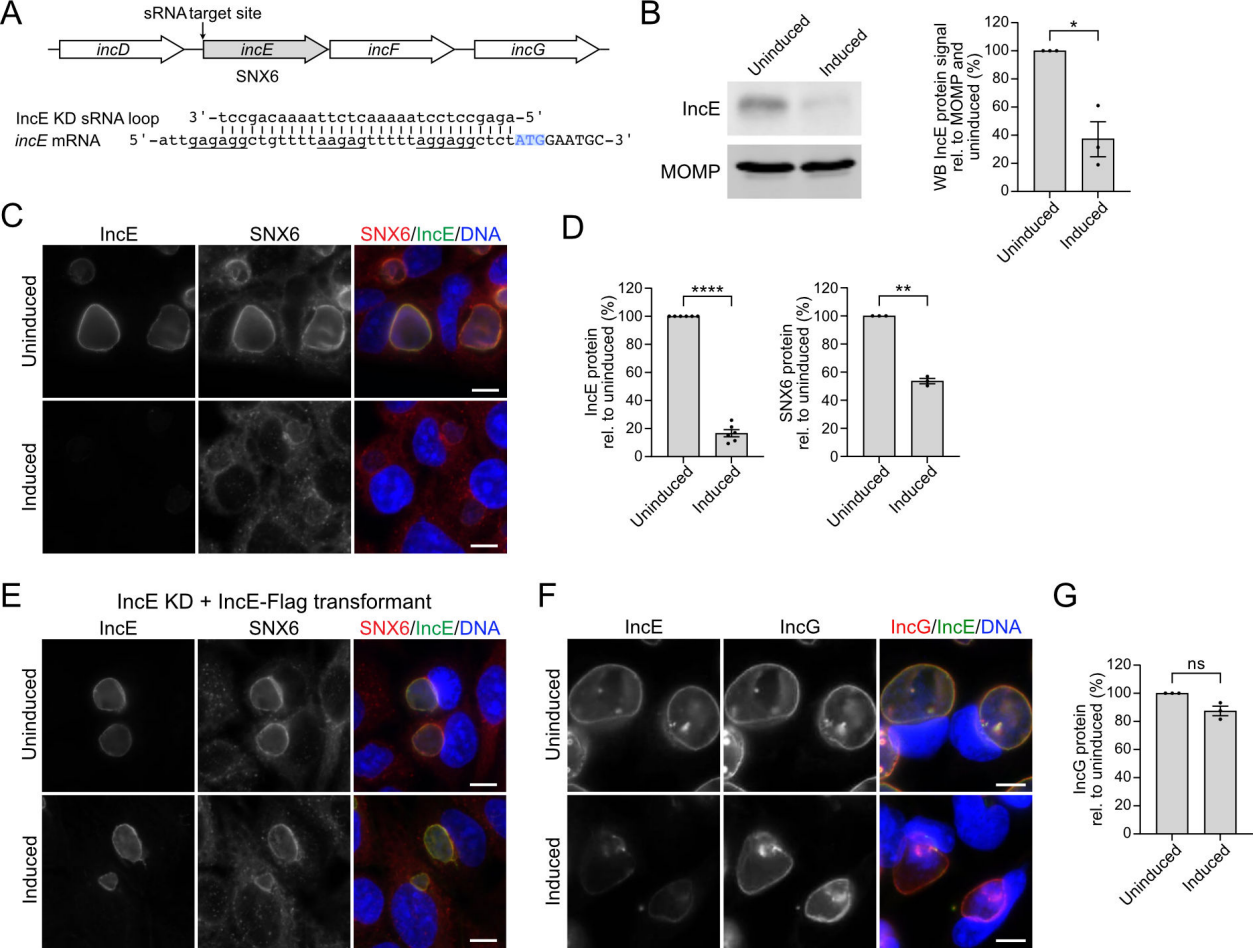
IncE knockdown does not cause polar effects on a downstream gene in the *incDEFG* operon. (**A**) Diagram of the *incDEFG* operon showing the site targeted by the IncE KD sRNA (top) and alignment of the engineered IncE KD sRNA and the *incE* mRNA (bottom). Three potential RBSs of *incE* are underlined, and the start codon is highlighted in blue. (**B**) Western blot of total cell lysates from HeLa cells infected with the *C. trachomatis* IncE KD transformant, either uninduced or induced with 1 ng/mL of aTc at 1 hpi and harvested at 24 hpi. IncE protein levels were detected with IncE-antibodies; MOMP serves as a loading control. A quantification of the IncE Western blot signal intensity, normalized to MOMP and the uninduced control, is also shown. (**C**) Immunofluorescence images of HeLa cells infected with the IncE KD sRNA transformant, uninduced or induced with 3 ng/mL of aTc at 1 hpi and stained at 24 hpi with antibodies to IncE (green) and SNX6 (red). (**D**) Quantification of the fluorescence intensity at the inclusion membrane for IncE (left) and SNX6 (right). (**E**) Immunofluorescence images of HeLa cells infected with the IncE KD + IncE Flag transformant, uninduced or induced with 3 ng/mL of aTc at 1 hpi and stained at 24 hpi with antibodies to IncE (green) and SNX6 (red). (**F**) Immunofluorescence images of HeLa cells infected with the IncE KD transformant and stained at 24 hpi with antibodies to IncE (green) and IncG (red). (**G**) Quantification of the fluorescence intensity of IncG. Data are shown as mean ± SEM from ≥three independent biological replicates, and from analysis of ≥150 inclusions per replicate and condition for immunofluorescence quantification. Unpaired, two-tailed Student’s *t*-test with Welch’s correction with *P < 0.05, **P < 0.01, and ****P < 0.0001; ns, not significant. DNA, as visualized with Hoechst 33342, is shown in blue. Scale bar is 10 µm. WB, Western blot; hpi, hours post infection; KD, knockdown.

We next tested if we could use our method to target a gene in an operon without causing polar effects on a neighboring gene. We separately knocked down the *C. trachomatis* inclusion membrane proteins IncE and IncG (34), which are encoded together on the *incDEFG* operon. Taking the same general approach as for IncA, we generated an IncE KD plasmid with a 30-nucleotide target sequence complementary to the 5′ untranslated region of IncE, directly upstream of the start codon and containing a potential RBS in the form of three closely positioned Shine Dalgarno-like sequences (Fig. 3A). Induction of IncE KD sRNA expression reduced IncE levels at 24 hpi by approximately threefold to 37% of an uninduced control by Western blots (Fig. 3B) and by sixfold to 17% ± 2% at the inclusion membrane, as measured with indirect immunofluorescence (Fig. 3C and D). We also found that IncE knockdown reduced inclusion membrane staining of host sorting nexin 6 (SNX6) (Fig. 3C and D), a known binding partner of IncE (35, 36). Inclusion membrane staining of IncE and SNX6 were both restored in a complemented strain in which we expressed Flag-tagged IncE together with the IncE KD sRNA (Fig. 3E; Fig. S3). Thus, this IncE knockdown data provide the first genetic evidence that IncE is necessary for SNX6 recruitment to the inclusion membrane. Importantly, IncE knockdown did not affect protein levels of IncG at the inclusion membrane (Fig. 3F and G), indicating that our method was able to knockdown IncE levels without causing polar effects on IncG protein abundance.

**Fig 4:**
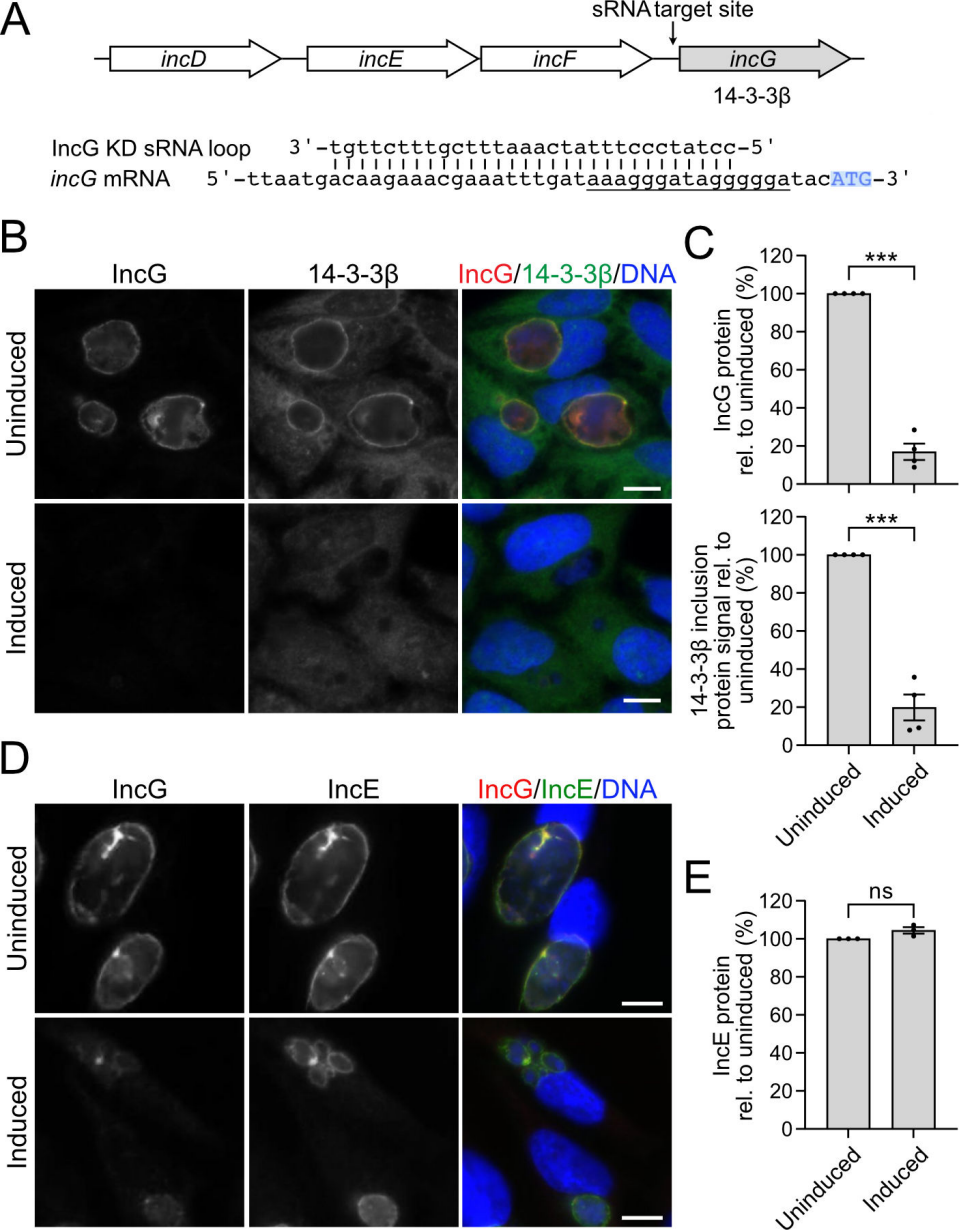
IncG knockdown does not cause polar effects on an upstream gene in the *incDEFG* operon (**A**) Diagram of the *incDEFG* operon showing the site targeted by the IncG KD sRNA (top) and alignment of the engineered IncG KD sRNA with *incG* mRNA (bottom). The potential RBS of *incG* is underlined, and the start codon is highlighted in blue. (**B**) Immunofluorescence images of HeLa cells infected with the IncG KD transformant stained at 24 hpi with antibodies to IncG (red) and 14-3-3β (green). (**C**) Quantification of the fluorescence intensity of IncG (top) and 14-3-3β (bottom). (**D**) Immunofluorescence images of HeLa cells infected with the IncG KD transformant and stained at 24 hpi with antibodies to IncG (red) and IncE (green). (**E**) Quantification of the fluorescence intensity of IncE. For all experiments, IncG knockdown was induced with 3 ng/mL of aTc at 1 hpi and compared to an uninduced control. Data are shown as mean ± SEM from ≥three independent biological replicates, from analysis of ≥150 inclusions per replicate and condition. Unpaired, two-tailed Student’s *t*-test with Welch’s correction with ***P < 0.001, ns, not significant. DNA, as visualized with Hoechst 33342, is shown in blue. Scale bar is 10 µm. hpi, hours post infection; KD, knockdown.

In a complementary set of experiments, we knocked down IncG and looked for effects on IncE. A KD sRNA expressing a targeting sequence for IncG (Fig. 4A) efficiently depleted both IncG and its host interacting protein 14-3-3β (37) from the inclusion membrane (Fig. 4B and C). IncG and 14-3-3β protein levels at the inclusion membrane were restored by co-expressing the IncG KD sRNA with exogenous Flag-tagged IncG (Fig. S4A and B). Thus, we induced functional knockdown of IncG and showed for the first time that IncG is necessary for 14-3-3β recruitment to the inclusion membrane. IncG knockdown did not affect IncE protein levels at the inclusion membrane (Fig. 4D and E). However, mRNA levels for IncG and IncE were both decreased by approximately 50% (Fig. S4C). These data show that IncG knockdown caused some degradation of the polycistronic *incDEFG* transcript but did not affect IncE protein levels.

These IncE and IncG knockdown studies demonstrate that our sRNA method was able to reduce protein levels of a targeted gene within the *incDEFG* operon without affecting protein levels of another gene in the same operon.

### Knockdown of a likely essential gene

**Fig 5:**
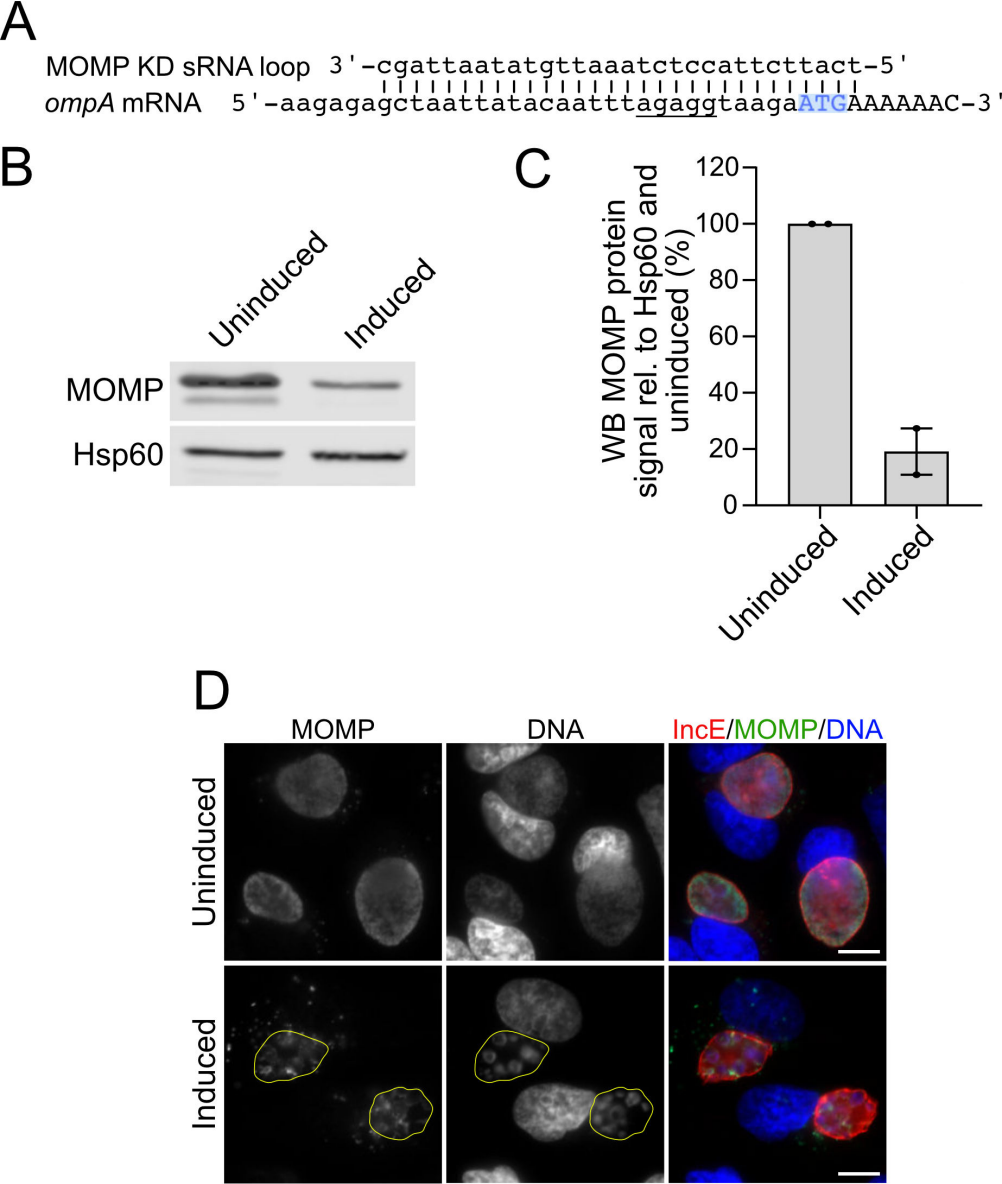
Knockdown of MOMP, which is encoded by the likely essential gene *ompA*. (**A**) Alignment of the engineered MOMP KD sRNA and the *ompA* mRNA. The putative RBS of *ompA* is underlined, and the start codon is highlighted in blue. (**B**) Western blot of total cell lysates from HeLa cells infected with the MOMP KD transformant and harvested at 24 hpi. MOMP protein levels were detected with a MOMP-specific antibody and Hsp60 and serve as loading controls for *Chlamydia*. (**C**) Quantification of the MOMP signal intensity normalized to Hsp60 and the uninduced control are shown. (**D**) Immunofluorescence images of HeLa cells infected with the MOMP KD transformant and stained at 24 hpi with antibodies to IncE (red) and MOMP (green). Inclusions in the induced samples are outlined with a yellow line. For all experiments, MOMP knockdown was induced with 3 ng/mL of aTc at 1 hpi. Data are shown as mean ± SEM from ≥two independent biological replicates. Two-way ANOVA with Sidak’s multiple comparisons test with ***P < 0.001. DNA, as visualized with Hoechst 33342, is shown in blue. Scale bar is 10 µm. hpi, hours post infection; KD, knockdown.

Finally, we tested if we could deplete a protein that is likely to be essential. We designed a knockdown sRNA for the MOMP, which is present in all *Chlamydia* isolates and has not been previously disrupted or knocked down. We generated a *C. trachomatis* transformant expressing a KD sRNA targeting the putative RBS and start codon of the MOMP gene, *ompA* (Fig. 5A). Western blot analysis showed that induction of the MOMP KD sRNA reduced MOMP protein levels by 5.3-fold compared to the uninduced control (Fig. 5B and C). Immunofluorescence microscopy after induction of the MOMP KD sRNA showed decreased MOMP staining of individual chlamydiae, compared to the uninduced control. There were no effects on IncE, which we used to visualize the chlamydial inclusion membrane (Fig. 5D). MOMP knockdown had deleterious effects on the *Chlamydia* infection, as shown by inclusions with few, enlarged RBs (Fig. 5D) that resembled aberrant bodies characteristic of chlamydial persistence (38–40), and a 16.3-fold decrease in infectious progeny at 32 hpi (Fig. S5). These results demonstrate that our sRNA knockdown method successfully depleted a conserved protein that is likely to be essential for the intracellular *Chlamydia* infection.

## DISCUSSION

In this study, we developed and used an sRNA-based method to knockdown four *C. trachomatis* proteins in a targeted manner. Depletion of IncA, IncG, and IncE caused a functional knockdown, as shown by production of a known deletion phenotype or disruption of the interaction with a specific host protein. We were able to knockdown an individual gene in the *incDEFG* operon without altering protein levels of a neighboring gene. In addition, by depleting MOMP protein levels and causing a severe progeny defect, we showed that our method can knockdown a presumed essential gene. Our *C. trachomatis* knockdown method has a number of favorable properties, including its conditional nature, which allows the timing and duration of knockdown to be controlled, and the ability to titrate and reverse the knockdown. In addition, the method and vector construction are simple and appears to have a high success rate because we were able to knock down all four genes that we targeted.

An issue with any knockdown method is its specificity. We incorporated a bioinformatic step in our target sequence selection to minimize off-target effects (Table S3). Additional measures could include optimizing target sequence length and induction conditions, such as using the lowest aTc concentration for good protein depletion, since off-target effects may be more likely with higher KD sRNA expression. The timing of KD sRNA expression could also be adjusted to match the endogenous expression pattern of the target mRNA. It will be important to use complementation to verify the specificity of a knockdown phenotype, as we did when we rescued the IncA multiple inclusion phenotype (Fig. 2B and C) and restored IncE-dependent SNX6 recruitment (Fig. 3E) and IncG-dependent 14-3-3β recruitment (Fig. S4A) to the inclusion membrane. This complementation approach could be improved by using a newly described riboswitch method (41) to control the expression of the complementing protein independently of the KD sRNA.

Our sRNA-based knockdown method has the potential to be widely used to study genes in *C. trachomatis* and to be applied to other *Chlamydia* spp. The main technical requirement is the ability to perform *Chlamydia* transformation, which has become a standard method in *C. trachomatis* (42, 43) and has recently been extended to the related species, *Chlamydia pneumoniae* (44), *Chlamydia psittaci* (45), and *Chlamydia muridarum* (46). Another requirement is the ability to verify protein depletion by measuring protein levels for the target. Most commonly, this means the availability of an antibody to detect the protein by Western blot analysis of a lysate of *C. trachomatis*-infected cells or with immunofluorescence analysis. RNA levels have been used to assess knockdown with the *Chlamydia* CRISPRi method (10), but may not reflect protein depletion because of differential transcript and protein stability.

Our sRNA-based knockdown approach adds to the growing repertoire of methods for gene disruption in *Chlamydia*. Genetic manipulation of *Chlamydia* and other obligate intracellular bacteria has historically been difficult because these organisms must be cultivated within eukaryotic host cells. In recent years there has been the welcome development of targeted gene knockout methods in *C. trachomatis*, including TargeTron insertional gene inactivation (28) and FRAEM allelic exchange (47, 48). However, the reduced genome of *Chlamydia* (5) makes it likely to have a high proportion of essential genes that cannot be permanently inactivated. Gene deletion may also select for compensatory mechanisms, including coding and regulatory mutations. A *C. trachomatis* conditional knockout method has recently been developed that pairs gene knockout with conditional depletion of a plasmid-expressed copy of the gene (7, 8).

Our sRNA-based knockdown approach and CRISPRi are complementary knockdown methods for *C. trachomatis* (9, 10, 49). Both methods can be used to study essential genes because they can knockdown a gene in a conditional manner. CRISPRi has recently been used in a multiplex approach to simultaneously knock down several protein target genes (13), and a similar approach could be taken to include multiple knockdown sRNAs, each for a different target, on the same plasmid. Both knockdown methods prevent expression of a target gene, but CRISPRi inhibits transcription by blocking the promoter, while our sRNA method targets translation. CRISPRi requires a PAM site close to the promoter and cannot selectively knockdown a gene in an operon because promoter inhibition affects transcription of all genes in the operon. In addition, CRISPRi uses a dCas enzyme, which has been associated with metabolic burden and toxicity issues (9, 50, 51). Our sRNA method may have a broader range of targets because of its potential to knockdown a gene in an operon without causing polar effects, as shown by separate knockdown of IncE and IncG in the *incDEFG* operon. We did not analyze the effects on IncD and IncF in the same operon because we did not have antibodies to these proteins. However, polar effects remain possible because sRNA–mRNA binding can promote degradation of the mRNA (52). Thus, when knocking down a gene in an operon with our sRNA-based method, it will be important to verify that there are no polar effects on neighboring genes. The number of *C. trachomatis* genes in an operon is not known, but the majority of genes in a bacterial genome are predicted to be in multi-gene operons (53). Consistent with this idea, Rudel and colleagues could only identify a putative promoter immediately upstream of the gene for 40% of the genes in the *C. trachomatis* genome (25), which suggests that a large proportion of chlamydial genes may be in operons.

This study points the way to wider use of this sRNA knockdown method in bacterial pathogens. Engineered sRNAs have been mainly used to deplete proteins in model bacteria such as *E. coli* (18–20, 22, 54). Recently, they have been used in combination with *Bacillus* Hfq as a targeted knockdown method in pathogenic Gram-positive bacteria (55). Our sRNA approach, which worked in the absence of Hfq (23), could be used for obligate intracellular bacteria that have a limited repertoire of genetic tools, including other *Chlamydia* spp., *Rickettsia*, *Coxiella*, *Ehrlichia*, and *Anaplasma* (56, 57). A conditional knockdown strategy is attractive for these bacteria, as well as *Mycoplasma* and *Treponema*, because they have small genomes as a result of genome reduction and are thus likely to have a higher proportion of essential genes.

In summary, our sRNA-based conditional knockdown method is a valuable addition to the *Chlamydia* genetic toolkit and may have a particular niche for studying the function of proteins encoded by an essential gene or an individual gene in an operon. The ability to knockdown a protein at a particular time and duration in the intracellular infection will be useful for studying individual steps in the developmental cycle, such as host cell entry, inclusion establishment, RB replication and RB-to-EB conversion. The broad impact of this knockdown method for investigating chlamydial gene function is shown by our knockdown results, which provide the first genetic proof that IncE is necessary for SNX6 recruitment to the inclusion membrane and that IncG is necessary for 14-3-3β recruitment. Furthermore, we generated the first strain in which MOMP can be conditionally depleted, which will allow the function of this abundant and immunodominant protein to be investigated in future studies. This sRNA knockdown strategy may be particularly useful for other pathogenic bacteria with small genomes and limited genetic tools.

## MATERIALS AND METHODS

### Antibodies used in this study

Primary antibodies used in this study are as follows: anti-MOMP (mouse, 1:30,000, gift from Ellena Peterson, University of California, Irvine), anti-IncA (rabbit, 1:2,000, gift from Dagmar Heuer, Robert Koch Institute, Berlin) for Western blot, anti-IncA (rabbit, 1:2,000) for immunofluorescence and anti-IncG (rabbit, 1:250) both gifts from Guangming Zhong, University of Texas Health Science Center, anti-Flag M2 (mouse, 1:200, F3165, Sigma-Aldrich), anti-14-3-3β clone K-19 (rabbit, 1:200, sc-629, Santa Cruz Biotechnologies), anti-IncE (rabbit, 1:1,000, gift from Joanne Engel, University of California, San Francisco), anti-SNX6 clone D-5 (mouse, 1:300, sc-365965, Santa Cruz Biotechnologies), anti-Hsp60/GroEL (mouse, 1:1,000, gift from Rick Morrison, University of Arkansas Medical Center), and anti-α-tubulin (rabbit, 1:1,000, ab18251, Abcam). Secondary antibodies for immunofluorescence staining were donkey anti-rabbit IgG AF488, donkey anti-rabbit IgG AF555, donkey anti-mouse IgG AF488, donkey anti-mouse IgG AF555, and goat anti-mouse AF647 (all 1:1,000, Invitrogen). Secondary antibodies for Western blot were goat anti-rabbit IRDye 680RD and goat anti-mouse IRDye 800CW (both 1:15,000, Li-Cor).

### Design of KD sequences

The 5′ untranslated region of the gene-of-interest within the *C. trachomatis* serovar L2 (434/Bu) genome was manually analyzed for potential ribosome binding sites (RBS), based on GA richness and resemblance to the canonical Shine–Dalgarno sequence. Two to three 30-nt sequences complementary to the putative RBS and/or start codon were selected and analyzed bioinformatically for target specificity using the TargetRNA3 web server (https://cs.wellesley.edu/~btjaden/TargetRNA3/) (30). We used the following parameters: candidate target probability of >0.2 and *P*-value of <0.05. The full-length KD sRNA was assembled by insertion of the 30-nt antisense sequence into the CtrR3 backbone and checked for correct folding using the RNAfold web server hosted by the ViennaRNA Web Services (http://rna.tbi.univie.ac.at/cgi-bin/RNAWebSuite/RNAfold.cgi) (58).

### Plasmid construction

All plasmids were generated using the NEBuilder HiFi DNA assembly mastermix (E2621, NEB) and subsequently transformed into *E. coli* strain DH5-alpha (NEB5-alpha). Plasmids were purified using the QIAprep Spin Miniprep kit (Qiagen) or Monarch Plasmid Miniprep kit (T1010S, NEB), followed by sequence verification via Sanger sequencing. Primer sequences can be found in Table S1 in the supplemental material. KD sequences were first subcloned into pRSETC-CtrR3 (24) using primers P1 + P2 to amplify the CtrR3 vector backbone and P3-6 to insert the respective KD sequence. pBOMB5-tet-KD plasmids were constructed using primers P7 + P8 and P9 + P10 to amplify the vector from pBOMB5-tet-CtrR3 (24), and primers P11 + P12 were used to amplify the CtrR3-KD sequence from the respective pRSETC-KD plasmids. Complementation plasmids pBOMB5-tet-IncA-KD + tet-mCherry and pBOMB5-tet-IncA-KD + tet-IncA-Flag were constructed using P7 + P13 and P10 + P14 to amplify the KD vector backbone from pBOMB5-tet-incA-KD plasmid. tetO_2_, a weakened tet promoter driving mCherry or IncA-Flag expression was amplified from pASKtet-GFP-L2 (gift from Scott Hefty, University of Kansas) (59) using P15 + P16. mCherry was amplified from pBOMB5-tet-mCherry (24) using P17 + P18, and incA-Flag was amplified from *C. trachomatis* serovar L2 genomic DNA using P19 + P20 while introducing silent mutations within the second and third codon to generate a knockdown-resistant version of IncA. Plasmids for incE, incG, and MOMP were generated by amplifying the KD vector backbone from the respective pBOMB5-tet-KD plasmid using P21 + P22 and P15 + P23. The incG and MOMP knockdown plasmids also contained tetR-mCherry, which was amplified from pBOMB5-tet-IncA-KD + tet-mCherry using P1 + P18. For incE/G complementation plasmids, tetR-Flag was amplified from pBOMB5-tet-IncA-KD + tet-IncA-Flag using P1 + P24, and incE or incG genes were amplified from *C. trachomatis* L2 genomic DNA using P25 + P26 or P27 + P28, respectively.

### Cell culture and *Chlamydia* transformation

HeLa (CCL-2, ATCC) and McCoy cells (CRL-1696, ATCC) were cultured at 37°C and 5% CO_2_ in Dulbecco's modified Eagle's medium (DMEM) (11995-065, Gibco) supplemented with 10% fetal bovine serum (FBS) (S11550, Atlanta Biologicals). Bacterial and mammalian cell cultures were routinely tested for Mycoplasma contamination. Transformation of *C. trachomatis* serovar L2 (ATCC, VR-902B) was performed as previously described (60). In brief, 1 × 10^7^ *Chlamydia* EBs were incubated with 12 µg of plasmid in 200 µL of CaCl_2_ buffer (50 mM CaCl_2_ in 10 mM TRIS, pH 7.4) for 30 min at room temperature, followed by spin infection of a six-well plate with a HeLa cell monolayer seeded the day before at 350,000 cells per well at 550 × *g* for 1 h in sucrose–phosphate–glutamic acid buffer (SPG; 200 mM sucrose, 20 mM sodium phosphate and 5 mM glutamate, pH 7.2). The inoculum was removed and replaced with DMEM + FBS. At 10–12 hpi, the medium was replaced with complete DMEM + FBS containing 10 µg/mL ampicillin (A9518, Sigma-Aldrich). At 48 hpi, infected host cells were disrupted using glass beads, with the collected *Chlamydia* being used to infect a new HeLa cell monolayer. After this second spin infection, the infected cells were incubated in complete DMEM + FBS containing 10 µg/mL of ampicillin and 1 µg/mL of cycloheximide (NC9651091, Chem Service Inc). This infection was labeled as passage 1 (P1). The last two steps were repeated until P3, resulting in a selected population of GFP-positive *Chlamydia* transformants with no visible aberrant bacteria. To obtain a clonal population, *Chlamydia* transformants from P3 underwent two rounds of plaque cloning in McCoy cells as previously described (61). Clonal *Chlamydia* transformants were scaled up by infection of HeLa cell monolayers, and EBs were separated from the host cell lysate by centrifugation at 250 × *g* for 20 min at 4°C, followed by crude EB concentration at 21,000 × *g* for 30 min at 4°C (JA-17 rotor, Beckmann Coulter Ultracentrifuge), and solubilization in SPG by ultrasonication. *Chlamydia* titers were determined to establish MOI by infection of HeLa cells with serial diluted EBs in SPG and manual counting of GFP-positive inclusion-forming units (IFU).

### *Chlamydia* infection and induction

Near-confluent HeLa cell monolayers grown on coverslips in 24-well dishes for immunofluorescence and Western blot experiments or in six-well plates for RT-qPCR analysis were infected in SPG at an MOI of 3 by centrifugation at 700 × *g* for 1 h at room temperature. After centrifugation, the inoculum was removed and replaced with DMEM + FBS. IncA KD and IncA KD + IncA Flag transformants were infected at a higher MOI of 5 to detect any multiple inclusion phenotype. For induction of sRNA, mCherry, and the complementation construct, infected cells were incubated with 3 ng/mL (6.5 nM) of anhydrotetracycline (aTc; 94664, Supelco) in DMEM + FBS at 1 hpi. Due to the short half-life of aTc at 37°C (62), aTc-containing medium was replenished at 16 hpi. For IncA washout experiments, aTc containing DMEM + FBS was removed at 18 hpi, infected cells were washed thrice in 1× PBS, and incubated for 4 and 6 h in DMEM + FBS without aTc.

### Immunofluorescence staining, imaging, and quantification

*Chlamydia-*infected HeLa cells, grown on glass coverslips in 24-well plates, were washed with 1× PBS and fixed with 100% ice-cold methanol for 10 min, followed by incubation in blocking buffer (2% FBS, 0.1% Saponin in 1× PBS) for 30 min at room temperature. After serial incubation with primary (overnight at 4°C) and secondary antibodies (2 h at room temperature), coverslips were washed in 1× PBS and mounted with ProLong Glass Antifade with NucBlue (Thermo Fisher, P36985). Immunofluorescence microscopy images were acquired using a ×63 objective (Plan-Apochromat, 1.4 Oil DIC, Zeiss) on an Axio Observer widefield fluorescence microscope, equipped with ApoTome.2, an Axiocam 506 mono camera, and a Colibri 7 LED light source (all Zeiss).

For quantification, fluorescence intensity of the entire inclusion, including the inclusion membrane, was determined by manual tracing of the inclusion membrane using the "raw spine contour” function, followed by measuring the mean intensity per area in the Zen software (Zeiss). A minimum of 10 different field of views and ≥120 inclusions were measured per biological and technical replicate. The background fluorescence was measured outside of the cell, subtracted from the inclusion fluorescence value, and the mean fluorescence intensity was calculated over all inclusions for each condition and replicated. Multiple inclusion phenotypes were quantified by manual counting of the number of inclusions per infected host cell from ≥10 different field of views and over 80 cells per biological and technical replicate. At least three biological and technical replicates were performed for each experiment. Color channels, brightness, and contrast of representative images shown in figures were adjusted equally within each experiment using ImageJ and Affinity designer software.

### Progeny assay

Progeny assays were performed as previously described (63). In brief, at 32 hpi, *Chlamydia-*infected HeLa cells were washed with 1× PBS and collected in SPG to harvest infectious EBs from the primary infection. Samples were subjected to one cycle of freeze–thaw to lyse the host cells, then serially diluted in SPG, and used to infect a new monolayer of HeLa cells in the absence of aTc. At 27 hpi, cells were fixed with ice-cold methanol for 10 min, followed by visualization of chlamydial inclusions with mouse anti-MOMP antibody and donkey anti mouse IgG A488. The number of inclusions, determined in 10 fields of view using a ×20 objective on an Axiovert 200M fluorescence microscope (Zeiss), was used to calculate the number of infectious progeny as IFU/mL. Progeny per host cell was determined by dividing IFU/mL by the number of host cells present at the time of infection, which was determined through counting trypsinized cells on a hemocytometer.

### Western blot

For IncA and MOMP, Cell lysates were prepared from infected cells from 1 well of a 24-well plate, washed with PBS followed by direct lysis in 2% SDS loading buffer with benzonase nuclease (Sigma), and heated at 95°C for 5 min. For IncE, infected cells from an entire six-well plate were washed with PBS and collected by scraping. Total cell lysates were prepared by lysing the cells in NP-40 lysis buffer (0.5% NP-40, 50 mM Tris-HCl [pH 7.4], 50 mM NaCl, 1 mM EDTA, and protease inhibitors) by light shaking for 20 min at 4°C, followed by centrifugation and addition of 2× SDS–loading buffer to the supernatant. Equal volumes of lysates were loaded and resolved by SDS-PAGE, followed by transfer onto nitrocellulose membrane (0.45 µm, Genesee Scientific). Membranes were blocked for 1 h in blocking buffer (5% bovine serum albumin [BSA] or 5% skim milk for IncE antibody, in 1× PBS containing 0.1% Tween 20), incubated with primary (18–24 h at 4°C) and secondary antibodies (1h at room temperature) in BSA blocking buffer, and imaged on an Odyssey CLx Imaging System (Li-Cor). MOMP band intensities were quantified using Image Studio Lite software (Li-Cor) and normalized to Hsp60 and then to the uninduced control.

### Statistical analysis

Microsoft Excel and Prism 9.4 software were used for data processing, plotting, and statistical analysis. Figures represent mean ± standard error of the mean (SEM) of at least three independent biological replicates, if not stated otherwise. Asterisks represent *P*-values obtained by either paired two-tailed Student’s *t*-test, unpaired two-tailed Student’s *t*-test with Welch’s correction, one-way ANOVA and Tukey’s multiple comparison test, or two-way ANOVA with Sidak’s multiple comparisons test using the mean values per biological replicate (*P < 0.05, **P < 0.01, ***P < 0.001, ****P < 0.0001).

## AFTER EPUB

*[This article was published on 13 November 2024 with errors in Fig. 1, 3, and S4. The figures were corrected in the current version, posted on 19 December 2024.]*

## SUPPLEMENTAL MATERIAL

Supplemental figures and tables mbio.02545-24-s0001.pdf Figures S1–S5 and Tables S1–S3.

ASM does not own the copyrights to Supplemental Material that may be linked to, or accessed through, an article. The authors have granted ASM a non-exclusive, world-wide license to publish the Supplemental Material files. Please contact the corresponding author directly for reuse.
