# Supplementary material for: Development of an sRNA-mediated conditional knockdown system for Chlamydia trachomatis
Source: mBio. 2024 Dec 13;16(2):e02545-24. doi: 10.1128/mbio.02545-24 (PMC11796381; doi:10.1128/mbio.02545-24)
Supplement: Supplemental figures and tables — Figures S1–S5 and Tables S1–S3. [file mbio.02545-24-s0001.pdf]

Supplementary figures

Figure S1

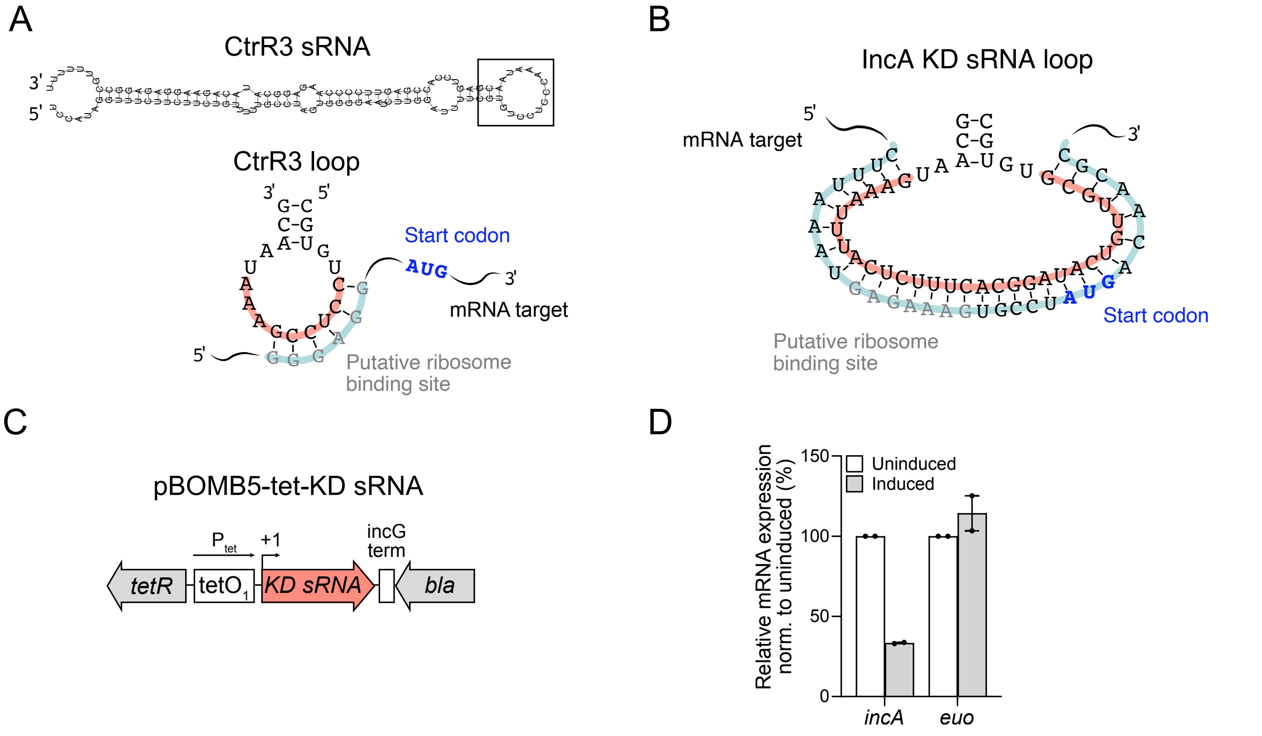

Supplementary Figure 1. Scheme of sRNA-mediated knockdown strategy and effect on *incA* mRNA levels. (A) Predicted folding of *C. trachomatis* CtrR3 sRNA using Vienna RNAfold. The 9 nucleotides loop region (shown in red) targeting the 5'-UTR of a target mRNA (in light blue) is shown in a box (top) and in magnification (bottom) (24). (B) Loop region (30 nucleotides) of the engineered IncA knockdown sRNA (IncA KD sRNA, shown in red) in which the CtrR3 targeting loop shown in (A) is replaced by a sequence that is complementary to the start codon, ribosome binding site and 5'-UTR of the *incA* mRNA (in light blue). (C) Scheme of the tetracycline-inducible expression cassette of the engineered KD sRNA in the pBOMB5 plasmid. +1 marks the transcription start site. (D) Quantification of *incA* and *euo* mRNA levels by RT-qPCR from HeLa cells infected with IncA KD transformant, uninduced or induced with 3 ng/mL aTc at 1 hpi and collected at 24 hpi. Data are shown as mean ± SEM from two independent biological replicates. hpi, hours post infection; KD, knockdown.

Figure S2

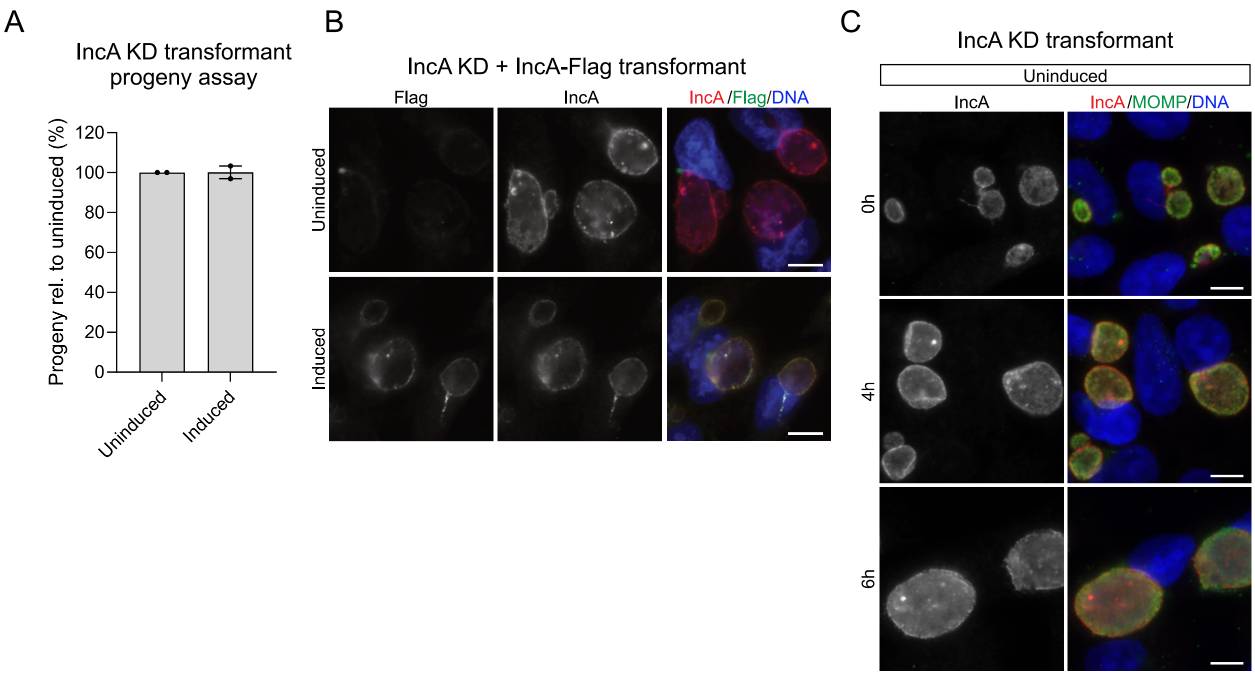

Supplementary Figure 2. Localization of overexpressed IncA-Flag and progeny assays with IncA KD transformant. (A) The number of infectious EBs produced by HeLa cells infected with the IncA KD transformant, uninduced or induced with 3 ng/mL aTc from 1 to 32 hpi was determined using progeny assays. (B) Immunofluorescence images of HeLa cells infected with the IncA KD + IncA-Flag transformant, uninduced or induced with 3 ng/mL aTc at 1 hpi and stained at 24 hpi with antibodies to IncA (red), and Flag (green). (C) Immunofluorescence images of HeLa cells infected with the IncA KD transformant. These infected cells were not induced, but stained at 18 (0h), 22 (4h) and 24 (6h) hpi with antibodies to IncA (red) and MOMP (green). DNA, as visualized with Hoechst 33342, is shown in blue. Scale bar is 10 μm. Data are shown as mean ± SEM from two independent biological replicates. hpi, hours post infection; KD, knockdown.

Figure S3

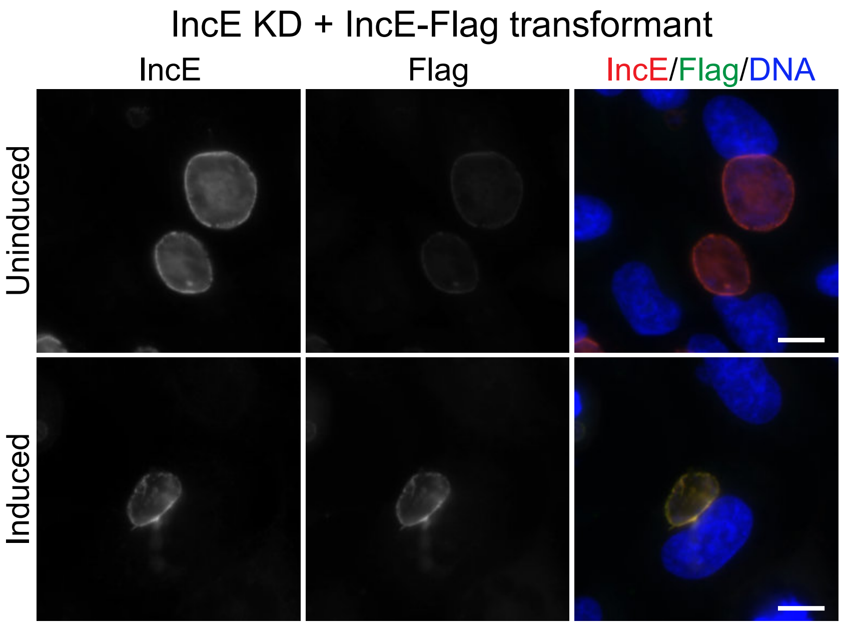

Supplementary Figure 3. IncE complementation. Immunofluorescence images of HeLa cells infected with the IncE KD + IncE-Flag transformant, uninduced or induced with 3 ng/mL aTc at 1 hpi and stained at 24 hpi with antibodies to IncE (green), and Flag (green). DNA, as visualized with Hoechst 33342, is shown in blue. Scale bar is 10 μm. hpi, hours post infection; KD, knockdown.

Figure S4

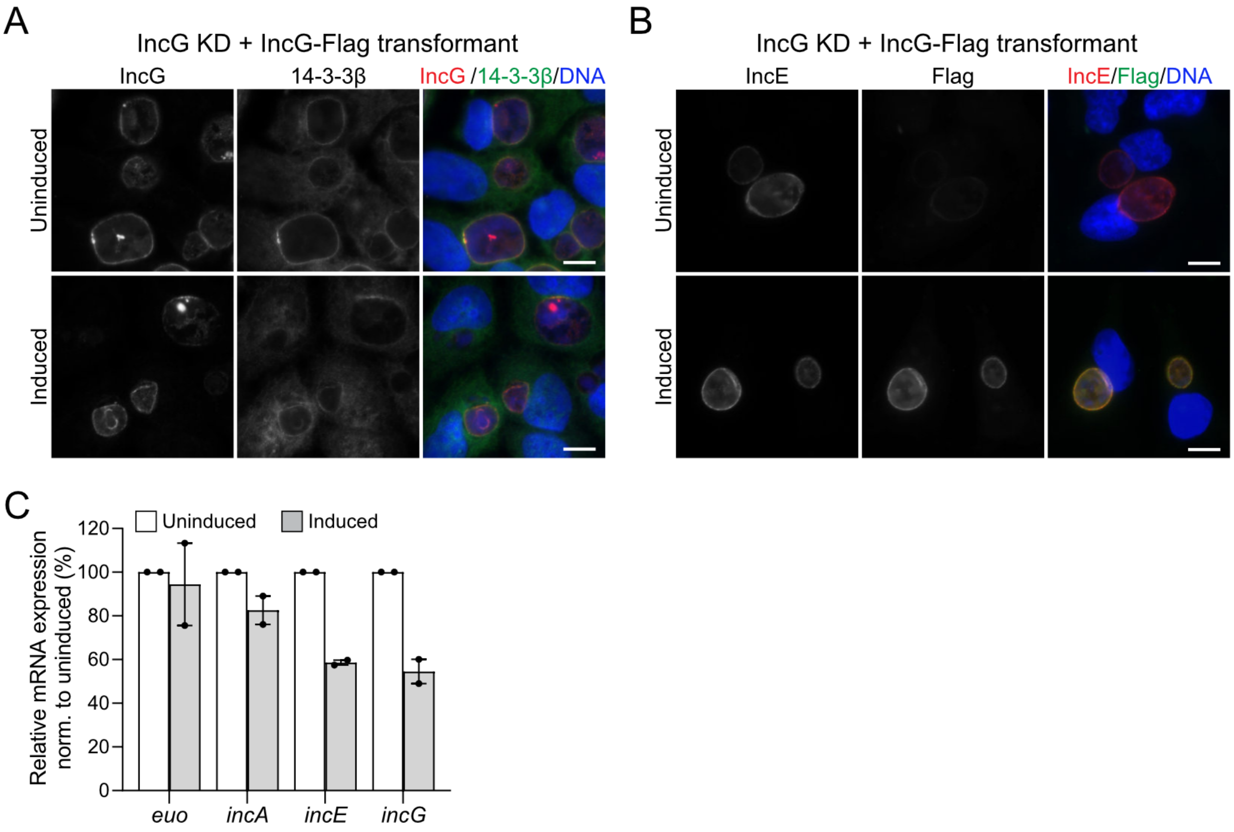

Supplementary Figure 4. IncG complementation and effect of IncG knockdown on the transcripts of genes in the operon. (A) Immunofluorescence images of HeLa cells infected with the IncG KD + IncG-Flag transformant, uninduced or induced with 3 ng/mL aTc at 1 hpi and stained at 24 hpi with antibodies to IncG (red), 14-3-3β (green) and (B) to IncE (red) and Flag (green). DNA, as visualized with Hoechst 33342, is shown in blue. Scale bar is 10 µm. (C) Quantification of *euo*, *incA*, *incG*, and *incE* mRNA levels by RT-qPCR from HeLa cells infected with the IncG KD + mCherry transformant, uninduced or induced with 3 ng/mL aTc at 1 hpi and collected at 24 hpi. Data are shown as mean ± SEM from two independent biological replicates. KD, knockdown; hpi, hours post infection.

Figure S5

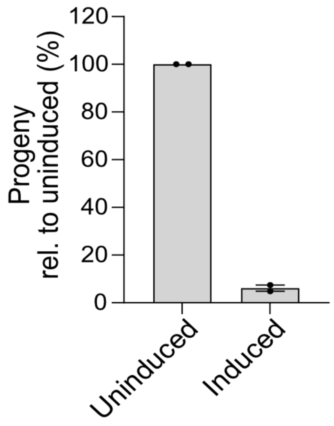

Supplementary Figure 5. Knockdown of MOMP causes a reduction in infectious progeny. Infectious progeny produced from HeLa cells infected with the MOMP KD transformant at 32 hpi are shown as percentage of the uninduced control. MOMP knockdown was induced with 3 ng/mL aTc at 1 hpi. Data are shown as mean ± SEM from two independent biological replicates. hpi, hours post infection; KD knockdown.

Supplementary Tables

Supplementary Table 1. Primer sequences for plasmid construction

#	Description	Sequence (5'-3')
P1	CtrR3-KD-F	acacgaaaatccaagattccc
P2	CtrR3-KD-R	taacgagtccaccgagttcc
P3	pRSET-incA KD	tcggtggactcgttactttaaatgagaaagtgcctatgacaacgcacacgaaaatccaa
P4	pRSET-incG KD	tcggtggactcgttaacaagaaacgaaatttgataaagggataggacacgaaaatccaa
P5	pRSET-incE KD	tcggtggactcgttaaggctgttttaagagtttttaggaggctctacacgaaaatccaa
P6	pRSET-ompA/MOMP KD	tcggtggactcgttagctaattatacaatttagaggtaagaatgaacacgaaaatccaa
P7	pBOMB5-vector-F	ggatagacattagctacagaatc
P8	pBOMB5-vector-incGterminator-R	gtaccaccggtggatccgtcgacgcg
P9	pBOMB5-vector-ptet-F	tggtaaaataactctatcaacgatagagtgtc
P10	pBOMB5-vector-R	gattctgtagctaatgtctatcc
P11	pBOMB5-CtrR3-KD-F	atccaccggtggtacgaatcggggaaaaatttcaataaaaaac
P12	pBOMB5-CtrR3-KD-R	agagttattttaccataggtttcttcttctgtttttgc
P13	pBOMB5-complement-vector-R	gccagcaaaaggccaggaac
P14	pBOMB5-complement-vector-F	ttgctcacatggaattcgatgc
P15	pBOMB5-complement-pASKtet-F	cataccggtatctccttcttaaag
P16	pBOMB5-complement-pASKtet-R	attccatgtgagcaaaattcctaagatctgttgactc
P17	pBOMB5-complement-mCherry-F	tggccttttgctggcttatttgtacagctcatccatgcc
P18	pBOMB5-complement-mCherry-R	ggagataccggtatggtctctaagggcgaggaag
P19	pBOMB5-complement-IncAFlag-F	tggccttttgctggcttacttatcgtcgtcatccttgtaatcggagctttttgtagagggtgatg

P20	pBOMB5-complement-IncA-R	gaaggagataccggtatgacgactcctactctaatcgtgactcctc
P21	pBOMB5-complement-vector2-F	ccataagcctctaagaattcag
P22	pBOMB5-complement-KD-R	ctagtggggaatcttggattttc
P23	pBOMB5-complement-vector2-R	ctgaattcttagaggcttatgg
P24	pBOMB5-complement-Flag-R	gattacaaggatgacgacgataag
P25	pBOMB5-complement-IncEFlag-F	cgtcgtcatccttgtaatcttgagttactaaaatcacttggtctg
P26	pBOMB5-complement-IncE-R	agaaggagataccggtatggaatgcgttaaacagttatgtag
P27	pBOMB5-complement-IncGFlag-F	cgtcgtcatccttgtaatcgaaggagcgtgatcgagaac
P28	pBOMB5-complement-IncG-R	agaaggagataccggtatgatctgttgtgacaaagtcttg

Supplementary Table 2. Primer sequences for RT-qPCR

Name	Sequence (5'-3')
incA_F	gcagggaatgctctttatctac
incA_R	cttgagatacagcagagagg
incE_F	cttgtcgagcgtacaatcaatg
incE_R	ggaagaacagcagcagtgc
incG_F	cgtacaatcaatgcctgttatag
incG_R	gcagatatggaagaacagcag
euo_F	ttattccgtgggacaagtgg
euo_R	tgcaagacttttccctttgc
16s rRNA_F	ggaaacggccgctaataccg
16s rRNA_R	gtaggcctttaccccaccaac
gapdh_F	tgcaccaccaactgcttagc
gapdh_R	ggcatggactgtggtcatgag

Supplementary Table 3. Bioinformatic analysis of potential off-targets of our targeting sequences

sRNA	Targets from TargetRNA3	Length of aligned sequence	Number of mismatches	Energy (kcal/mol)	Probability	p-value
incA	***incA***	**30**	**0**	**-43.15**	**0.62**	**2.40E-13**
	pmpD	7	0	-8.2	0.37	1.10E-08
	secD	9	1	-7.51	0.26	4.20E-06
	rpoC	22	3	-10.7	0.23	2.20E-05
incE	***incE***	**30**	**0**	**-42.77**	**0.62**	**1.20E-12**
	pmpD	10	1	-6.91	0.36	5.20E-08
	secD	6	0	-7.04	0.25	1.20E-05
	rpoC	12	2	-10.34	0.23	4.00E-05
	CTL_RS01075	17	1	-21.64	0.2	2.10E-04
	CTL_RS02110	6	0	-9.62	0.2	2.20E-04
incG	***incG***	**30**	**0**	**-44.99**	**0.63**	**9.70E-13**
	pmpD	19	5	-7.54	0.36	6.10E-08
	secD	11	2	-7.33	0.25	1.60E-05
	rpoC	26	11	-9.13	0.24	3.00E-05
	cdsD	25	5	-22.77	0.2	2.80E-04
MOMP	***MOMP***	**30**	**0**	**-41.97**	**0.79**	**8.90E-16**
	pmpD	13	3	-7.35	0.36	3.20E-08
	rpoC	10	2	-5.97	0.26	5.10E-06
